# Spectral and Angular Characteristics of the High-Contrast Dielectric Grating under the Resonant Interaction of a Plane Wave and a Gaussian Beam

**DOI:** 10.3390/ma15103529

**Published:** 2022-05-14

**Authors:** Stefano Bellucci, Volodymyr Fitio, Tatiana Smirnova, Iryna Yaremchuk, Oleksandr Vernyhor, Yaroslav Bobitski

**Affiliations:** 1 INFN-Laboratori Nazionali di Frascati, Via E. Fermi 54, 00044 Frascati, Italy; 2 Department of Photonics, Lviv Polytechnic National University, S. Bandera Str. 12, 79013 Lviv, Ukraine; iryna.y.yaremchuk@lpnu.ua (I.Y.); vernyhor@gmail.com (O.V.); yaroslav.v.bobytskyi@lpnu.ua (Y.B.); 3 Institute of Physics of NAS of Ukraine, Prospect Nauky, 46, 03028 Kyiv, Ukraine; smirnova@iop.kiev.ua; 4 Department of Physics, University of Rzeszow, Pigonia Str. 1, 35959 Rzeszow, Poland

**Keywords:** diffraction dielectric grating, resonance, finite cross-section beam, rigorous coupled wave analysis (RCWA), sampling theorem, discrete Fourier transform

## Abstract

The resonant interaction of a plane wave and a one-dimensional Gaussian beam with a high-contrast dielectric grating was analyzed. Rigorous coupled wave analysis (RCWA) was used to numerically model the diffraction of a plane wave by the grating. RCWA, a discrete Fourier transform at the fulfillment (of the conditions) of the sampling theorem, was used to study diffraction of the Gaussian beam. The grating can be considered as a one-dimensional photonic crystal along which the waveguide mode propagates under resonance. The corresponding photonic crystal has both allowed and forbidden photonic bands for the propagating waveguide mode under resonance due to the high-contrast dielectric permittivity. There is no significant difference between the spectral and angular characteristics under the interaction of the plane wave or the Gaussian beam with grating, if the waveguide mode is in the forbidden photonic bandgap. The reflection coefficient from the grating is practically equal to unity for both cases. Resonant spectral and angular characteristics become wider at the Gaussian beam diffraction compared to the resonance curves for the plane wave in the case when the waveguide mode is in the allowed photon bandgap. The reflection coefficient from the grating becomes less than unity and its value tends to unity when the Gaussian beam width increases.

## 1. Introduction

In recent years, intensive studies of refractive index sensors based on the dielectric grating on dielectric substrates have been carried out [1,2,3,4]. These sensors are based on the waveguide mode resonance [5,6,7] by the dielectric gratings on the dielectric substrates. The reflection coefficient from a periodic structure is equal to unity under the resonant interaction of an incident plane wave and grating [8,9,10]. The operation principle of such sensors is based on a change in the resonant wavelength of the incident wave or the resonant angle of the beam incidence by the gratings with the change in the refractive index of the studied surrounding medium [3,10,11].

The predominantly numerical studies of the diffraction of a plane wave by the grating were carried out using rigorous coupled wave analysis (RCWA) [12] with numerically stable algorithms [13,14,15]. The RCWA is asymptotically accurate [13] and converges faster than the other methods for dielectric periodic structures. It is used to analyze various periodic structures [16,17], including photonic crystals [18].

However, the results obtained using RCWA for the resonant interaction of a plane wave with a dielectric grating often did not coincide with the experimental data [2,19]. It is obvious that the grating was irradiated not by a plane wave but by a finite cross-section beam in the experiments. It should be noted that the resonant wavelengths, experimentally determined and numerically predicted, coincide with satisfactory accuracy. At the same time, the reflection coefficients determined experimentally are significantly less than unity, but significantly higher than the values (R>0.1), which can be explained by Fresnel reflection. Simultaneously, the widths of the experimentally measured spectral resonance curves increased. This discrepancy between the numerically predicted and experimental results can be explained by the following reasons:

(a) there is no strict repeatability of the groove shape and the period of the grating in the real grating, which was especially important in [19];

(b) the grating was irradiated by a finite cross-section beam during the experimental studies. The beam width can be much less than the distance over which the resonant waveguide mode propagates into the gratings. It is important for a slight refractive index modulation of the grating medium [2,16]. Therefore, it is important to numerically study the interaction of a finite cross-section beam with the dielectric grating, under waveguide resonance.

This problem has been analyzed in a relatively small number of scientific works. Apparently, this is due to the fact that the result of the diffraction of a finite cross-section beam by the grating almost coincides with the result of the diffraction of the plane wave in the absence of resonance [20].

The studies of the diffraction of the finite size beam from three to 20 periods of using the finite-difference frequency-domain method were presented in [21]. The influence of beams limited in transverse size by the rigorous boundary element method was studied using the rigorous boundary element method in [22]. According to the simple scalar theory of diffraction, it was explained that the angular anomaly of the reflected beam from the grating is a direct result of the finite size of the beam [23]. In [24], a study of beam diffraction using a metal grating of finite size, under excitation of the surface plasmon–polariton resonances, was carried out. Theoretical modeling suggests an expansion of the resonances when the grating size decreases. The resonant filter for telecommunications based on gratings finite in size has been developed, which is tuned due to the change in the incidence angle of the beam by the gratings [25]. It was shown in [26] that the fields of Gaussian beams scattered by reflective gratings differ markedly from those predicted by geometrical considerations. A corrected theory of diffraction [27] by a finite volume grating, which is rather complicated for practical use, was proposed. The influence of the finite size of the incident Gaussian beam on the spectrum of anomalous reflection and the shape of the energy distribution in the reflected beam from the waveguide with the grating was analyzed using the developed approximate theory [28]. The authors of [29] attempted to extend the RCWA method for a grating with a finite number of periods using supercells. Guizal et al. [30] developed a method called aperiodic RCWA, in which the permittivity of the finite grating is represented by a Fourier integral leading to an integral–differential equation. Lalanne and coworkers [31,32] introduced the use of absorbing boundary conditions and ideal layer matching at the unit cell ends, to numerically analyze the finite periodic structures. The simplest and rather effective theory of the reflection of the finite size beam from the grating was given in [33], where the solution was provided in the analytical form. This theory consists of decomposing the limited beam into plane waves. The direct and inverse transformations are used in the analysis. This theory does not imply a search for the spatial distribution of the amplitude and, accordingly, the power of the wave passes through the gratings. Therefore, it is impossible to check whether the law of energy conservation is fulfilled during diffraction by the purely phase grating. Further development of the theory of limited light beam diffraction by the dielectric grating was presented in [34]. The method is based on the representation of such a beam as an expansion into plane waves using the Fourier transform. Then, the amplitude reflection and transmission coefficients of RCWA are determined for each plane wave. The field distribution of the reflected and transmitted beams is determined by the inverse discrete Fourier transform [35]. The number of plane waves into which a limited light beam is decomposed must correspond to the sampling theorem [36,37]. The developed method [34] corresponds to the energy conservation law, if the grating is dielectric [38] at the diffraction of the finite cross section beam. Thus, the sum of the powers of reflected and transmitted beams is equal to the power of the incident beam on the grating. Results of studies of finite beam diffraction by non-absorbing gratings obtained by the holographic method using the photosensitive media were obtained using this method [20,34]. Such gratings are characterized by the low modulation of the grating medium refractive index. In [20], diffraction analysis of the one-dimensional Gaussian beam and the beam described by the rect(x) [35] function was performed. Experimental data [20] of the reflection coefficient dependence on the beam width coincided well with the corresponding dependence obtained by the numerical method, according to the developed theory.

A sensor based on a relief dielectric grating with a rectangular bar cross-section was characterized by unique properties [4]. The lower refractive index was 1.333 (the refractive index of the tested medium), and the higher refractive index was 2.0 at the fill factor F=0.5 in this grating. It turned out that such a structure had some unexpected properties. The full width at half maximum (FWHM) decreased sharply (approximately from 20 nm to 0.15 nm) [4] at almost constant sensitivity S~250 nm/RIUU and accordingly increased the figure of merit (FOM), which can be defined as FOM = S/FWHM [39] for certain grating parameters (grating period and thickness, wavelength). The FOM increased from 14 to 1620. Such features of this periodic structure were explained in [40], in which an analysis of diffraction was performed using numerical methods for the plane wave and the Gaussian beam. A comparison of the obtained results allowed us to conclude that the grating under the waveguide mode resonance, with respect to the high reflection coefficient from the grating, should be considered as a one-dimensional photonic crystal [41]. Such a high-contrast photonic crystal can have both forbidden and allowed photonic bandgaps [41,42]. There is no bandgap [41] in the case of low dielectric contrast in the photonic crystal. Thus, such sensors will have a high FOM [2,20,34].

It was found that FOMs are equal in wavelength and angle, both for a plane wave [2] and for the limited beam in cross-section [20,34] for sensors based on the holographic gratings (small modulation of the grating medium refractive index). In addition, it was shown that FWHM for the plane wave is uniquely related by the analytical expression linking wavelength, grating period, beam angle, and wavelength attenuation constant of waveguide mode at the propagation by the grating [20]. Moreover, the constant attenuation was determined at the grating irradiation by the Gaussian beam. However, it is not certain that these provisions will be valid for gratings with high-contrast changes in dielectric constant over a period. Allowed and forbidden photonic bandgaps are possible in such gratings in one-dimensional photonic crystals. Therefore, it is desirable to continue numerous studies in its irradiation with the plane wave and the Gaussian beam. These studies should be aimed at obtaining the spectral and angular dependences of the reflection coefficient from the grating, with various parameters (L, d, θ, λ), as well as the changes in the resonant wavelength and the resonant angle, upon changes in the surrounding medium refractive index. According to the results of these studies, it is important to determine the angular and spectral sensitivity, as well as FWHM and FOM, and how they depend on the Gaussian beam width L, for the allowed and forbidden photonic bandgaps.

## 2. Results of Numerical Modeling and Discussions

**Figure 1:**
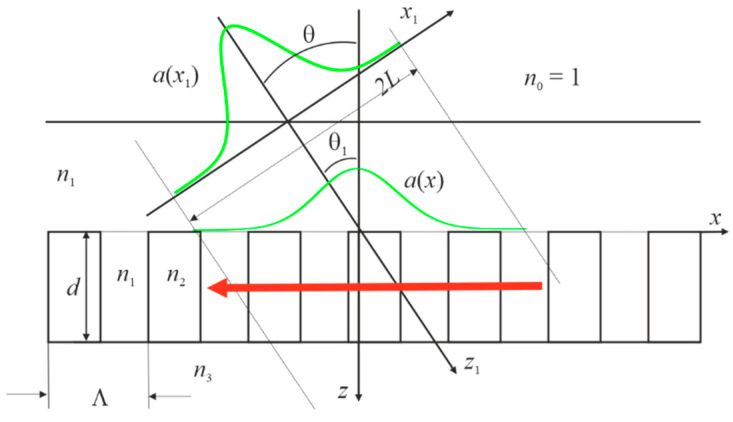
Incidence of the Gaussian beam on the grating with the following parameters: Λ=0.697748 μm, $n_{1}=1.333,\;n_{2}=2,\;n_{3}=1.45,$ F=0.5. Angle θ is 0 or π/18 according to numerical analysis. Wavelength λ and grating thickness d are constant.

The method described in detail in [20,34,40] was used to analyze the diffraction of the Gaussian beam by the dielectric grating. The notation of physical quantities in this paper is the same as in [40]. The researched periodic structure with the corresponding symbols is shown in Figure 1. The red arrow in the figure schematically shows the waveguide mode propagating from right to left, which loses its energy when interacting with the grating; as a result, the reflected and missed beams are formed.

**Figure 2:**
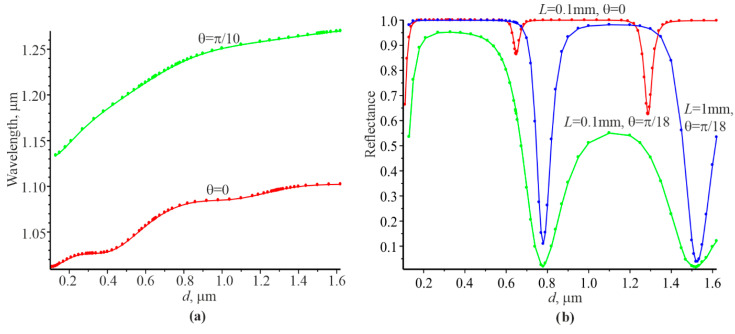
Dependences of the resonant wavelength on the grating thickness for the plane wave (**a**). Dependence of the relative reflectance $P_{r}$ on the grating for the Gaussian beam (**b**). L is the half-width of the Gaussian beam in accordance with Figure 1. The distribution of the Gaussian beam per 1001 plane wave (reprinted with permission from ref. [40]) was used for numerical calculations.

The graphical materials (Figure 2) and the corresponding numerical data given in [40] were used for our study.

Each grating thickness d has its own resonant wavelength $\lambda_{rez}$ at which the reflection coefficient is equal to unity, as follows from Figure 2a. It can be seen that the resonant wavelengths are higher than at the normal incidence of the plane wave at the incidence angle of the plane wave = π/18. Figure 2b indicates that the reflection coefficient $P_{r}$ takes on minimum values at certain thicknesses corresponding to $\lambda_{rez}$ according to Figure 2a: d=0.65 μm and d=1.288 μm for θ=0 and d=0.78 μm and d=1.52 μm for θ=π/18. Therefore, it can be argued that the corresponding $\lambda_{rez}$ values are in the photonic allowed bandgap according to the theory of photonic crystals. At the same time, reflection coefficient $P_{r}$ is practically equal to unity for a wide range of thicknesses d, even for the Gaussian beam width L=0.1 mm. That is, these thicknesses and the corresponding resonant wavelengths are in the forbidden photonic bandgap.

**Figure 3:**
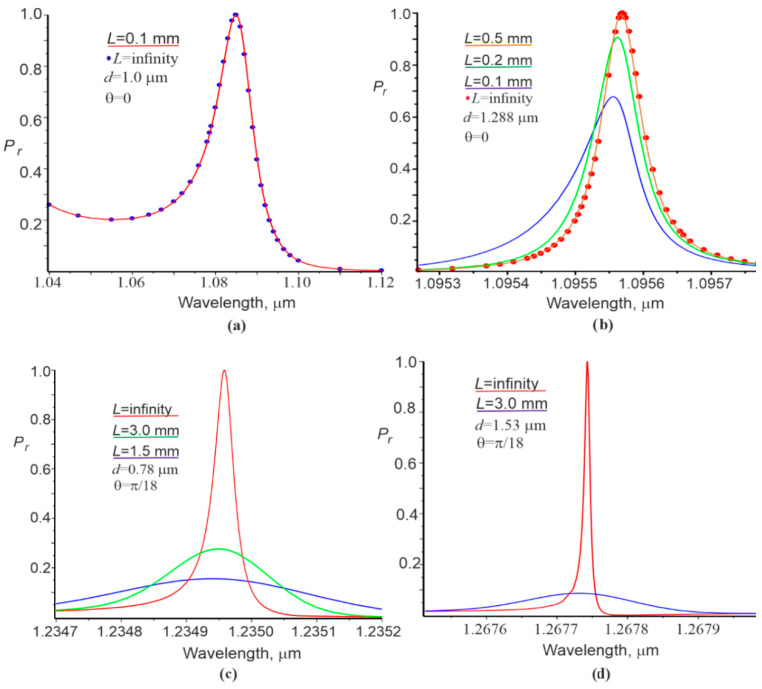
Spectral dependences of the reflection coefficient from the grating for the plane wave (L=infinity) and the Gaussian beam. The color of the curves corresponds to the color of straight segments under the value of L: (**a**) forbidden photon bandgap; (**b**–**d**) allowed photon bandgap.

The spectral dependences of the reflection coefficient on the grating are shown in Figure 3. The reflection spectrum for the Gaussian beam, i.e., the solid red curve, coincides with the blue circles, corresponding to the plane wave at the thickness d=1 μm (Figure 3a, photonic bandgap).

The spectral curves under conditions when the waveguide mode is in the allowed photon bandgap and can propagate a considerable distance in the grating are shown in the Figure 3b–d.

It can be concluded that the reflection coefficient $P_{r}\left(\lambda \right)$ increases when L increases and approaches the spectral curve at L=∞ (Figure 3b). There is also a clear correlation between the reflection coefficient at resonance for the Gaussian beam in accordance with Figure 2a and the width of the spectral curve for the plane wave. That is, a smaller reflection coefficient $P_{r}$ for a Gaussian beam results in a smaller width of the spectral curve for the plane wave.

The widths of the spectral resonance curves at the level of 0.5 at the incidence of the plane wave and the Gaussian beam (δλ ≡ FWHM) were determined using Figure 3. The attenuation indices γ at the propagation of the resonant waveguide mode in the grating were determined for the linear sections of the curves $\ln\left|r_{0}\left(x\right)\right|$. These data are presented in Table 1 (columns 4 and 5, respectively).

**Figure 4:**
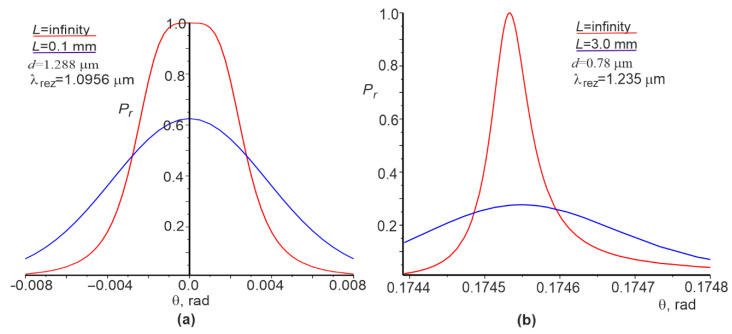
Angular dependences of the reflection coefficient $P_{r}$ at normal incidence (**a**) and the incidence angle of π/18 (**b**).

The angular dependences of the reflection coefficient for the plane wave and the Gaussian beam are shown in Figure 4. It can be seen that angular dependence has a somewhat flat vertex (Figure 4a, red curve) at the normal incidence of the plane wave. However, it is absent for other cases. The widths of the angular dependences δθ are given in column 6 of Table 1. There is a clear correlation between the widths δλ and δθ for the plane wave and the attenuation index γ; a smaller γ results in a narrower δλ and δθ for the plane wave.

**Figure 5:**
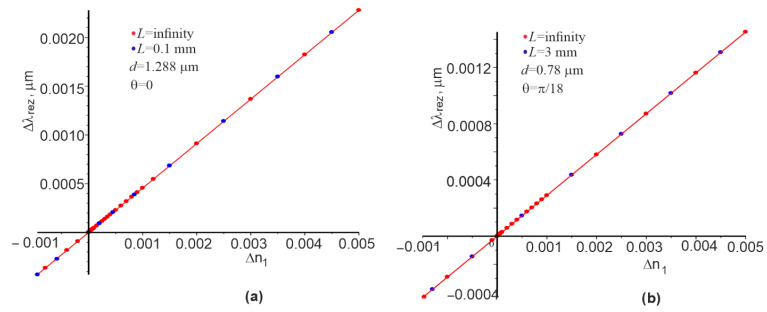
Dependence of $\Delta \lambda_{rez}$ on the change of refractive index $\Delta n_{1}$ for the Gaussian beam and the plane wave at the normal incidence of the Gaussian beam and the plane wave (**a**), and for the Gaussian beam and the plane wave incidence at the angle of π/18 (**b**). Straight red lines are drawn between the two extreme points corresponding to the minimum and maximum value of $n_{1}$.

The dependences of the change in the resonant wavelength $\Delta \lambda_{rez}$ on the change in the refractive index $\Delta n_{1}$ for the Gaussian beam and the plane wave at other constant parameters are also interesting. The corresponding dependencies are shown in Figure 5. These dependences are linear in nature, and they can be used to determine the spectral sensitivity $S_{\lambda }=\Delta \lambda_{rez}/\Delta n_{1}$. It can be argued that the sensitivities $S_{\lambda }$ are the same for the Gaussian beam and the plane wave since the red and green circles lie on the same straight line (see Figure 5). This is consistent with the findings in [34], where the same result was obtained but for a low modulation dielectric constant of the grating medium. It can be concluded on the basis of the data in Table 1 that $S_{\lambda }$ values are slightly larger at the normal incidence of the Gaussian beam or plane wave compared to the angle of incidence π/18.

The spectral sensitivities $S_{\lambda }$ for some cases are shown in Table 1, column 7. Knowing $S_{\lambda }$ and δλ, we determined $\mathrm{FOM}=S_{\lambda }/\delta \lambda$, as presented in Table 1 (column 8). It can be seen that FOMs are mostly larger among the studied cases at the beam angle of incidence on the grating of π/18.

**Figure 6:**
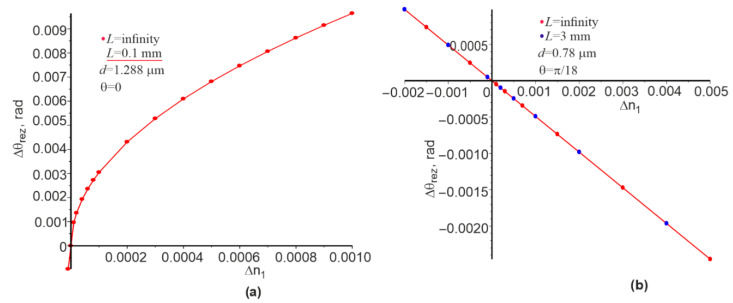
Dependence of $\Delta \theta_{rez}$ on the change in refractive index $\Delta n_{1}$ for the Gaussian beam and the plane wave at the normal incidence of the Gaussian beam and the plane wave (**a**), and for the Gaussian beam and the plane wave incidence at the angle of π/18 (**b**). A straight red line is drawn between the two extreme points corresponding to the minimum and maximum value of $n_{1}$.

The dependences of the change in the resonance angle $\Delta \theta_{rez}$ on the change in the refractive index $\Delta n_{1}$ for the Gaussian beam and the plane wave at other constant parameters are shown in Figure 6. The nature of the corresponding curves significantly depends on the initial value of the angle. If the angle of incidence of the beams at $n_{1}=1.333$ is zero, then the corresponding dependence is nonlinear (Figure 6a). If the angle of incidence of the beams is equal to π/18, then the corresponding dependence is linear in the range of $\Delta n_{1}$ from −0.002 to 0.005, allowing us to calculate $S_{\theta }=-\Delta \theta /\Delta n_{1}$. Results of the calculation of $S_{\theta }$ for the angle of π/18 are included in column 9 of Table 1. The ratio ${\mathrm{FOM}}_{\theta }=S_{\theta }/\delta \theta$ can be calculated knowing the values $S_{\theta }$, which are given in column 10 of Table 1. It can be expressed that

(1) $$S_{\lambda }/\delta \lambda \approx S_{\theta }/\delta \theta .$$

Equation (1) was obtained in [11] analytically for dielectric gratings based on photopolymer compositions, which are characterized by insignificant modulation of the refractive index of the grating medium. Equation (1) is also true for sensors based on a prism structure and based on metal gratings on the metal substrate in which surface plasmon–polariton waves are excited [11]. Moreover, Equation (1) is valid both for the plane wave (columns 8 and 10, lines 1 and 3) and for the cross-section limited beam (columns 8 and 10, lines 2 and 4). However, we can consider the $S_{\theta }$ at the beam incidence angle on the grating significantly different from zero, as evidenced by Figure 6. Therefore, some of the cells of Table 1 are not filled for cases where the normal incidence of the plane wave or the Gaussian beam is on the grating.

It follows from Table 1 (columns 8 and 10) that the FOM in the transition from the plane wave to the Gaussian beam decreases several times due to increasing δλ and δθ at constant sensitivities $S_{\lambda }$ and $S_{\theta }$ (columns 7 and 9, respectively). However, the FOM is large enough at the L shown in column 11, except for the data of rows 9 and 10, which correspond to the photon bandgap. In this case, the FOM is very small (41.7) due to the high value of δλ=11.5 nm.

Analytical expressions defining δλ and δθ for the plane wave for gratings with small modulation of the refractive index through Λ, λ, θ, and especially γ, which is determined for the Gaussian beam on the linear part of the $\ln\left|r_{0}\left(x\right)\right|$ dependence, were presented in [33]. The corresponding equations are as follows:

(2) $$\delta \lambda \approx \frac{\lambda \Lambda \gamma }{2\pi }.$$

(3) $$\delta \theta \approx \frac{\lambda \gamma }{2\pi \cos\theta }.$$

Numerical experiments have confirmed the validity of these equations for three-dimensional phase gratings with low modulation of the grating medium refractive index [20,34]. However, these relations are not fulfilled for our case when $n_{1}=1.333$ and $n_{2}=2.0$. Here, δλ=0.037 nm, δθ=0.0624 mrad, and δθ=0.0624 mrad according to row 1 of Table 1 and δλ=0.021 nm and δθ=0.031 mrad according to Equations (2) and (3).

However, Equations (2) and (3) can be useful in another aspect. If certain grating parameters ($n_{1},\;n_{2}$, F, Λ, wavelength, and beam incident angle on the grating) correspond to a certain reflection coefficient, then the reflection coefficient does not change upon changing the wavelength λ, period Λ, grating thickness d, or beam width L K times. The value of K can be either larger or smaller than one. This statement is true for both the plane wave (L=∞) and the beam of width L. However, numerical experiments have shown that γ will change 1/K times. Therefore, we can assume that, when the parameters change K times, δλ will also change K times, and δθ will be unchanged in accordance with Equations (2) and (3). This was confirmed by our numerical experiments for data rows 1–8 of Table 1. Numerical experiments also showed that $S_{\lambda }$ also will change K times, while $S_{\theta }$ will remain unchanged. Therefore, the FOM with such a change in parameters will remain unchanged, which is consistent with Equation (1).

The obtained relations can be called rules of similarity. They can be useful in determining the grating parameters to obtain the resonance at the certain wavelength generated by the laser, if the resonance conditions are known at the specific wavelength. Accordingly, the coefficient of change K will be equal to the ratio of the two wavelengths.

## 3. Conclusions

The obtained numerical results confirm the concept of the resonant waveguide mode propagation in the grating of a one-dimensional photonic crystal.

The values of FWHM for the plane wave and the Gaussian beam (rows 9 and 10, column 4 of Table 1) are the same and relatively wide (Figure 3a) if the waveguide mode is in the forbidden photon bandgap. Otherwise, when the waveguide mode is within the allowed photon bandgap, FWHM decreases with increasing L and approaches FWHM for the plane wave.

Numerical studies have shown that the dependences $\lambda_{rez}$ and $\theta_{rez}$ are linear to change $n_{1}$ (see Figure 5b and Figure 6b) at the beam incidence angle of θ=π/18 rad. However, $\theta_{rez}$ is nonlinear on $n_{1}$ at the initial angle θ=0 (see Figure 6a). The corresponding sensitivities $S_{\lambda }$ and $S_{\theta }$, as well as FOM for both cases, which satisfy Equation (1), can be calculated on the basis of these linear dependences. However, Equations (2) and (3) are not valid for a large contrast of changes in the dielectric permittivity in the grating. Nevertheless, the right and left parts of these relations are within the same order of magnitude; in this particular case, they differ by about two times.

It is shown that the similarity rule is also valid for the limited beam in width. According to this rule, the reflection coefficient will not change when the wavelength λ, the period Λ, the grating thickness d, and the beam width L change K times. Therefore, δλ will change K times and δθ will remain unchanged, while $S_{\lambda }$ will also change K times and $S_{\theta }$ will remain unchanged. Therefore, the FOM will remain unchanged with such a change in parameters, which is consistent with Equation (1).

## Figures and Tables

**Table 1:** Parameters of the grating (columns 1,2) and beam (column 11), as well as the results of numerical analysis (columns 3–10).

Parameters	d, μm	θ, rad	$\lambda_{rez},\;\mu m$	δλ, nm	$\gamma ,\;mm^{-1}$	δθ, mrad	$S_{\lambda },\;nm$	$S_{\lambda }/\delta \lambda$	$S_{\theta },\mathrm{rad}$	$S_{\theta }/\delta \theta$	L, mm
No	1	2	3	4	5	6	7	8	9	10	11
1	0.78	π/18	1.23496	0.037	-	0.0624	291	7865	0.491	7869	∞
2	0.78	π/18	1.23496	0.186	0.1540	0.321	291	1556	0.491	1544	3.0
3	1.52	π/18	1.26754	0.01186	-	0.01944	300.1	25,304	0.492	25,309	∞
4	1.52	π/18	1.26754	0.0595	0.04709	0.09763	300.1	5044	0.492	5039	10
5	1.288	0.0	1.09557	0.071	-	0.00543	456	6423	-	-	∞
6	1.288	0.0	1.09557	0.101	11.059	0.0090	456	4515	-	-	0.1
7	0.65	0.0	1.06449	0.24	-	-	405	1688	-	-	∞
8	0.65	0.0	1.06449	0.28	22.07	-	405	1446	-	-	0.1
9	1.0	0.0	1.08490	11.5	-	-	480	41.7	-	-	∞
10	1.0	0.0	1.08490	11.5	-	-	480	41.7	-	-	0.1

## Data Availability

Data are available by the corresponding authors, upon reasonable request.
